# Therapeutic Effects of Esomeprazole on Pancreatic and Lung Injury in Acute Pancreatitis: An Experimental Study

**DOI:** 10.3390/medicina61020200

**Published:** 2025-01-23

**Authors:** Yusuf İskender Tandoğan, Oktay Aydin, Faruk Pehlivanli, Kuzey Aydinuraz, Çağatay Erden Daphan, İlker Kaplan

**Affiliations:** 1 Department of General Surgery, Gaziantep State Hospital, Gaziantep 27470, Turkey; 2 Department of General Surgery, Kirikkale University School of Medicine, Kirikkale 71450, Turkeykuzeyaydinuraz@yahoo.com (K.A.);; 3 Department of General Surgery, Ermenek State Hospital, Karaman 70400, Turkey; lkrkpln@gmail.com

**Keywords:** acute pancreatitis, esomeprazole, cerulein, inflammation

## Abstract

*Background and Objectives*: During acute pancreatitis, leakage of pancreatic enzymes into the gland results in autolysis of the pancreas. The lungs are also involved in this process. This study aimed to investigate the therapeutic effects of esomeprazole on damaged pancreatic tissue and affected lung tissue in rats with acute pancreatitis. *Materials and Methods*: The 24 Wistar-Albino male rats were divided into three groups: Control group (2 mL 0.9% saline solution was given intraperitoneally, n = 8); PCT group (acute pancreatitis was induced and then 2 mL 0.9% saline solution was administered intraperitoneally, n = 8); ESM group (acute pancreatitis was induced and then 10 mg/kg esomeprazole was administered intraperitoneally, n = 8). Then, the lungs and pancreas were completely removed, and blood samples were taken from all rats for histopathological and biochemical examination. *Results*: Pancreatic edema, vacuolization, necrosis, and inflammation in the PCT group were higher than in the control and ESM groups. Alveolar edema, alveolar distension, alveolar PMNL infiltration, and alveolar wall thickness in the PCT group were higher than in the control and ESM groups. Furthermore, IL-β (F = 40.137, *p* < 0.001), TNF-α (F = 40.132, *p* < 0.001), MIP-2 (X^2^ = 19.245, *p* < 0.001), ICAM-1 (F = 14.312, *p* < 0.001), NO (F = 25.873, *p* < 0. 001), amylase (F = 30.333, *p* < 0.001), and lipase (X^2^ = 16.141, *p* < 0.001) values measured in serum were different among groups. Pairwise group comparisons revealed that IL-β, TNF-α, MIP-2, and amylase levels in the ESM group were lower than in the PCT group (*p* < 0.05). *Conclusions*: Esomeprazole could be recommended in clinical practice during acute pancreatitis treatment due to its therapeutic effects on damaged pancreatic and lung tissues secondary to pancreatitis in rats.

## 1. Introduction

Acute pancreatitis is a clinically and histologically regressive acute inflammation of the pancreas developed by the self-digestion of pancreatic tissue after activating pancreatic enzymes [1]. Gallstone obstructions, alcohol, drug toxicities, and infections are common in etiology. Clinical findings of acute pancreatitis often start with vague abdominal pain and may manifest as electrolyte disturbances, hypotension, acid–base balance disorder, and sepsis. Although the clinical course is mild, local, regional, and systemic complications that may result in mortality can develop in the acute and subacute stages [2]. The most common extra-abdominal complication is acute lung injury and associated acute respiratory distress syndrome (ARDS). ARDS accounts for most of the mortality due to pancreatitis in the acute period [3]. There are supportive treatments, surgical treatments, or drug therapies for etiologic factors in treating acute pancreatitis [4]. Pharmacologic agents (such as somatostatin, ulinastatin) continue to be tried in drug treatment [5,6].

Esomeprazole, a gastric proton pump inhibitor, is the S-isomer of omeprazole and is used in the treatment of pathological gastric hypersecretory conditions, including erosive esophagitis, gastroesophageal reflux disease, peptic ulcer disease, *Helicobacter pylori* eradication, relief of non-steroidal anti-inflammatory drug side effects and Zollinger–Ellison Syndrome. In addition, esomeprazole has been reported to have anti-inflammatory, antioxidant, anti-inflammatory, antifibrotic, and antiproliferative effects on sepsis, acute lung injury, lung fibrosis, dermal injury, renal ischemia–reperfusion injury, and hepatocellular fibrosis [7,8,9,10,11,12,13,14,15].

Since experimental studies have shown that esomeprazole has anti-inflammatory and antifibrotic effects in inflammatory and other lung injuries, its therapeutic effects on acute pancreatitis have not been evaluated in the literature. This experimental study aimed to investigate the therapeutic effects of esomeprazole on both damaged pancreatic tissue and affected lung tissue in rats with cerulein-induced acute pancreatitis.

## 2. Materials and Methods

This experimental study was conducted after approval by the Institutional Experimental Animal Ethics Committee (Decision number: 46, Decision date: 24 December 2020).

### 2.1. Animals

In the study, 24 Wistar-Albino male rats weighing between 220 and 280 g were randomly divided into 3 groups as follows:

– Control group (2 mL of 0.9% NaCl solution was given intraperitoneally, n = 8);
– PCT group (acute pancreatitis was induced by cerulein, and then 2 mL 0.9% saline solution was administered intraperitoneally, n = 8);
– ESM group (acute pancreatitis was induced by cerulein, and then 10 mg esomeprazole was administered intraperitoneally, n = 8).

### 2.2. Experimental Model

**Figure 1:**
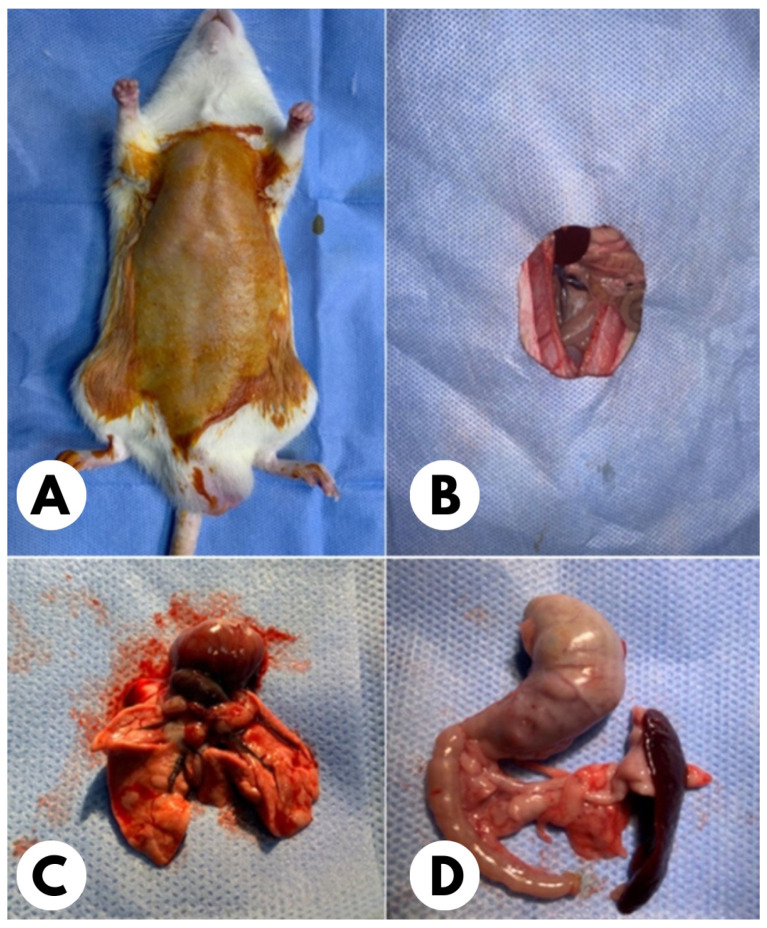
After rats were sacrificed, they were shaved and disinfected with povidone–iodine before the operation (**A**); laparotomy was performed through a midline incision (**B**), and the lung (**C**) and pancreas with stomach, duodenum, and spleen were completely removed (**D**).

Acute pancreatitis was induced by intraperitoneal injection of 50 μg/kg/h cerulein diluted in 0.5 mL saline four times at one-hour intervals in rats except the control group. Twelve hours after acute pancreatitis was induced, 2 mL of saline solution was intraperitoneally administered to rats in the PCT group, and a single dose of 10 mg esomeprazole was intraperitoneally administered to rats in the ESM group [16,17,18]. Twelve hours after this procedure (i.e., 24 h after the last injection of the cerulein), 50 mg/kg ketamine (Ketalar^®^, Parke Davis, Pinto, CA, USA) and 10 mg/kg xylazine hydrochloride (Rhompon^®^, Bayer, Leverkusen, Germany) were administered to all rats intramuscularly and then, through the midline abdominal incision, the abdomen was entered, and pancreatic tissue was resected completely. Then, a thoracotomy was performed, and the lungs were resected totally (Figure 1). Blood was collected through the abdominal aorta for biochemical examination. After samplings, the rats were sacrificed by cervical dislocation [19].

### 2.3. Histopathological Evaluation

**Figure 2:**
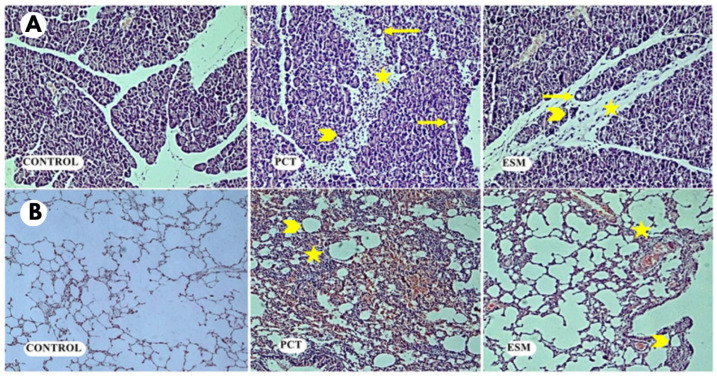
Histopathologic examination results showed that edema, vacuolization (arrow), necrosis (arrowhead), and inflammation (star) in pancreatic tissue were higher in the PCT group compared to the ESM group. In the control group, these findings were absent (**A**). In addition, alveolar edema, alveolar distension (arrowhead), alveolar PMNL infiltration (star), and alveolar wall thickness in the lung tissues of the PCT group increased much more than in the ESM group. In the control group, these findings were absent (**B**) (H&E, ×100, ×400).

The tissues were fixed in 10% formaldehyde and embedded in paraffin blocks. Then, sections taken from the tissues were stained with hematoxylin–eosin (H&E) and examined under a light microscope. The edema, inflammation, vacuolization, and necrosis levels in pancreatic tissue and alveolar edema, alveolar distension, alveolar wall thickness, and PMNL infiltration levels in the lung tissues were scored using a 0–4 points scale (0 = no, 1 = minimal, 2 = mild, 3 = moderate, 4 = severe) (Figure 2) [20].

### 2.4. Biochemical Analysis

Nitric oxide from iNOS is an important factor underlying the systemic and local hemodynamic disorders and oxidative tissue damage associated with acute pancreatitis [21]. Biochemical measurements of MPO activity in the pancreas, lung, and other tissues have been used as markers of neutrophil sequestration [22]. To analyze these molecules, the lung and pancreatic tissue samples were homogenized separately with PBS (Phosphate Buffer Saline, pH: 7.4) solution, and streptolysin-O (SLO) (Streptolysin O Enzyme, catalog no: SEN0167, Enzyme-Sunlong Biotech Co., Ltd., Hangzhou, China) (HU/mL), myeloperoxidase (MPO) levels (Rat Myeloperoxidase catalog number: SL 1170Ra, Enzyme-Sunlong Biotech Co., Ltd., Hangzhou, China) (U/mg), and nitric oxide (Rat Nitric Oxide, catalog number: SL0531Ra, Enzyme-Sunlong Biotech Co., Ltd., Hangzhou, China) (μmol/L) were measured using a spectrophotometer (Thermo Scientific Multiskan FC, 2011-06, Waltham, MA, USA).

In addition, TNF-α and IL-1β cytokines are the main proinflammatory cytokines produced by macrophages. The increase in these cytokines in acute pancreatitis and acute lung injury due to pancreatitis also correlates with the severity of inflammation [1]. MIP-2 (macrophage inflammatory protein) is secreted by monocytes and neutrophils. It is one of the chemokines responsible for chemotaxis in inflammation [23]. ICAM-1 (intercellular adhesion molecule) is a transmembrane protein that enables neutrophils to adhere to the endothelial surface. ICAM-1 is activated in inflammation and plays a role in neutrophil-mediated lung injury [24]. Serum amylase and lipase levels are among the first and most frequently preferred parameters in diagnosing acute pancreatitis. To analyze these molecules, blood samples were centrifuged at 3000 rpm for 10 min, and interleukin-1beta (IL-1β) (Rat Interleukin 1beta, catalog no: SL0402Ra, Enzyme-Sunlong Biotech Co., Ltd., Hangzhou, China) (pg/mL), tumor necrosis factor-alpha (TNF-α) (Rat Tumor Necrosis Factor α, catalog number: SL0722Ra, Enzyme-Sunlong Biotech Co., Ltd., Hangzhou, China) (pg/mL), macrophage inflammatory protein-2 (MIP-2) (Rat Macrophage Inflammatory Protein 2, catalog number: SL0465Ra, Enzyme-Sunlong Biotech Co., Ltd., Hangzhou, China) (pg/mL), intercellular adhesion molecule-1 (ICAM-1) (Rat Intercellular Adhesion Molecule 1, catalog number: SL0382Ra, Enzyme-Sunlong Biotech Co., Ltd., Hangzhou, China) (ng/mL), amylase (U/L), and lipase (U/L) levels were measured using a (Roche^®,^ Basel, Switzerland) spectrophotometer (Thermo Scientific Multiskan FC, 2011-06, Waltham, MA, USA).

### 2.5. Statistical Analysis

The Shapiro–Wilks test was used to test the normal distribution of the study’s results. The One-Way Analysis of Variance (ANOVA) test was used to compare parametric data, and the Tukey Multiple Comparisons test was used in post hoc analysis (*p* < 0.05). The Kruskal–Wallis test was used to compare the nonparametric data, and the Mann–Whitney U test was used in post hoc analysis (*p* < 0.05).

## 3. Results

### 3.1. Histopathologic Examination

Edema, vacuolization, necrosis, and inflammation in pancreatic tissue were higher in the PCT group compared to the control group and the ESM group. In addition, edema, vacuolization, necrosis, and inflammation were higher in the ESM group than in the control group. These findings were absent in the control group (Figure 2).

**Figure 3:**
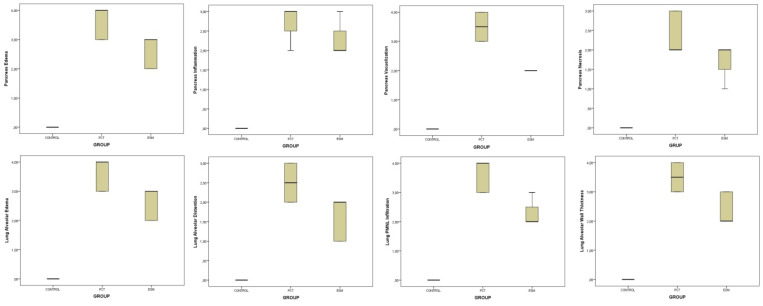
Each graph shows the histopathological examination score results of the pancreas and lung tissue of the treatment groups.

As a result of histopathological scoring, pancreatic edema (X^2^ = 19.413, *p* < 0.001), inflammation (X^2^ = 18.688, *p* < 0.001), vacuolization (X^2^ = 21.445, *p* < 0.001), and necrosis (X^2^ = 19.413, *p* < 0.001) scores were statistically different among the groups (Table 1). Pairwise group comparisons revealed that in the pancreatic tissue, edema, vacuolization, and necrosis level scores were different between the groups (*p* < 0.05). However, the level of inflammation in the pancreatic tissue was statistically different between the control group and the PCT group and between the control group and the ESM group, but not different between the PCT group and the ESM group (Table 2, Figure 3).

Lung injury developed in all rats in the PCT group, resulting in alveolar edema, alveolar distension, alveolar PMNL infiltration, and increased alveolar wall thickness. Similar changes were observed in the ESM group, but they were less severe than in the PCT group. In the control group, these findings were almost absent (Figure 2). Histopathologic scoring revealed that alveolar edema (X^2^ = 20.034, *p* < 0.001), alveolar distension (X^2^ = 19.642, *p* < 0.001), alveolar wall thickness (X^2^ = 20.236, *p* < 0.001), and alveolar PMNL infiltration (X^2^ = 21.018, *p* < 0.001) were statistically different between all groups (Table 1). Pairwise group comparisons showed that alveolar edema, distension, wall thickness, and PMNL infiltration score values in the lung after pancreatitis were different among the groups (*p* < 0.05) (Table 2, Figure 3).

### 3.2. Biochemical Analysis Results

**Figure 4:**
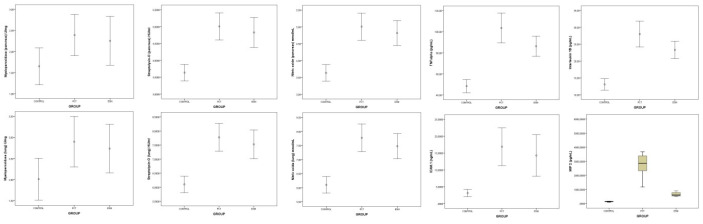
Graph of biochemical analysis results of the groups.

Biochemical analysis of lung and pancreatic tissue samples revealed that streptolysin-O levels measured in pancreatic (F = 24.516, *p* < 0.001) and lung (F = 24.886, *p* < 0.001) tissues were different among the groups. MPO levels measured in lung tissue were also found to be different among the groups (F = 4.008, *p* = 0.034). Furthermore, tissue NO levels in the pancreas (F = 25.873, *p* < 0. 001) and lung (F = 25.144, *p* < 0. 001) were different among the groups (Table 3). Pairwise group comparisons revealed that streptolysin-O and NO levels measured in pancreatic and lung tissues were different between the control group and PCT group and between the control group and ESM group, but they were not different between the PCT group and ESM group (Table 4, Figure 4).

**Figure 5:**
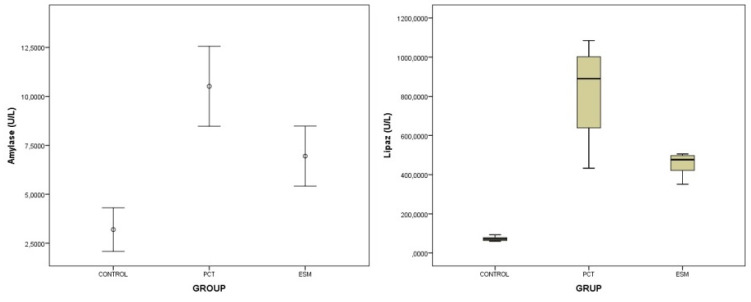
Graph of serum amylase and lipase levels of the groups.

On the other hand, the IL-β (F = 40.137, *p* < 0.001), TNF-α (F = 40.132, *p* < 0.001), MIP-2 (X^2^ = 19.245, *p* < 0.001), ICAM-1 (F = 14.312, *p* < 0.001), amylase (F = 30.333, *p* < 0.001), and lipase (X^2^ = 16.141, *p* < 0.001) values were different among the groups (Table 3, Figure 4, Figure 5). Pairwise group comparisons revealed that ICAM-1 and lipase levels measured in serum were different between the control group and the PCT group and between the control group and the ESM group, but not between the PCT group and the ESM group. However, IL-β, TNF-α, MIP-2, and amylase levels were found to be different among the three groups (Table 4).

## 4. Discussion

Acute pancreatitis is an inflammation that begins with the leakage of pancreatic enzymes into the gland and results in autolysis of the pancreas. Acute pancreatitis may rarely lead to pancreatic necrosis, abscess, gastrointestinal bleeding, sepsis, and death. On the other hand, the most common extra-abdominal involvement of acute pancreatitis is in the lungs, which can be seen in different forms ranging from simple dyspnea to a highly mortal clinic such as ARDS. Acute lung injury is the most common complication causing mortality in the early period of pancreatitis, but the mechanism of action is still not fully elucidated in the literature [2].

Esomeprazole magnesium trihydrate is still widely used for the treatment of peptic ulcers and other related diseases. Studies have reported that esomeprazole can reduce the inflammatory and fibrotic responses in an acute lung injury model. It has also been reported that esomeprazole controls inflammation by suppressing the expression of proinflammatory molecules, such as vascular cell adhesion molecule-1, inducible nitric oxide synthase, TNF-α, IL-1β, and IL-6. In addition, its antioxidant effect is associated with strong induction of heme oxygenase-1 (HO1), and its antifibrotic effect is related to the inhibition of fibroblast proliferation and down-regulation of profibrotic proteins, including receptors for transforming growth factor β (TGFβ), fibronectin, and matrix metalloproteinases (MMPs) [12,13].

At the end of our study, histopathological evaluation results revealed that acute pancreatitis developed in the PCT and ESM groups in the first 24 h compared to the control group. On the other hand, administration of esomeprazole significantly decreased pancreatitis-induced edema, vacuolization, and necrosis in the pancreas, but its effect on inflammation was not statistically significant. In addition, alveolar edema, distension, alveolar PMNL infiltration, and alveolar wall thickness in the lung were significantly decreased after esomeprazole administration. These findings suggest that esomeprazole administration could reduce the damage secondary to acute pancreatitis in both pancreatic and lung tissues.

The biochemical analysis results of our study showed that esomeprazole administration did not change streptolysin-O and MPO levels in damaged pancreas and lung tissues. On the contrary, esomeprazole administration significantly decreased serum IL-β, TNF-α, amylase, and MIP-2 levels. Thus, it was concluded that esomeprazole could reduce serum levels of IL-β and TNF-α cytokines released from macrophages by reducing macrophage activation in rats with acute pancreatitis, showing anti-inflammatory activity and reducing the destructive effects of acute pancreatitis.

Frossard et al. reported that lung injury followed pancreatitis, MIP-2 concentration in the lung peaked 12 h before the pancreatic concentration, and serum MIP-2 concentration was significantly associated with the formation of pulmonary leakage [23]. It has also been suggested in the literature that MIP-2 may be a good marker for developing lung injury, and this molecule has a strong activator effect on neutrophils [25,26,27]. Our study showed that MIP-2 levels in the PCT group were considerably higher than in healthy rats. In addition, serum MIP-2 levels of the subjects in the ESM group were found to be quite low and close to the values of healthy rats. Thus, it was thought that esomeprazole caused a significant decrease in MIP-2 levels secreted by monocytes and neutrophils, and this effect could be shown by decreasing neutrophil activities. These findings suggested that esomeprazole could show anti-inflammatory effects in acute pancreatitis and lung damage.

Under stable conditions, ICAM-1 is continuously expressed at low levels in endothelial and epithelial cells or on the surface of alveolar cells. This molecule modulates cell recognition, activation, proliferation, differentiation, and motility, thus helping to stabilize the body’s internal environment [24]. During inflammation, ICAM-1 plays an important role in pathological events associated with inflammatory reactions, including acute renal failure and acute pancreatitis. However, it has been reported that the devastating effects of acute pancreatitis and associated lung injury occur less frequently in ICAM-1 knockout mice [28,29]. In our study, we observed a significant numerical decrease in ICAM-1 level after esomeprazole administration, which provides adhesion of neutrophils to the endothelial surface and is involved in neutrophil-mediated lung injury; however, this decrease was not statistically significant. Therefore, we thought that esomeprazole did not affect ICAM-1 levels. However, we argued that a statistically significant reduction in serum ICAM-1 levels could be obtained if esomeprazole activity was observed for longer than 24 h.

Oxidative stress developing in the early stage of acute pancreatitis may cause oxidative damage in pancreatic tissue by leading to lipid peroxidation in pancreatic acinar cells and the formation of large amounts of reactive oxygen species [30]. In our study, although esomeprazole was found to have an anti-inflammatory effect by decreasing acute phase reactants, it was thought that it could not decrease serum NO levels and, therefore, could not show antioxidant activity.

On the other hand, esomeprazole significantly decreased the elevated serum amylase levels in acute pancreatitis but was not statistically effective on serum lipase levels. However, when the numerical data were analyzed, it was observed that esomeprazole also significantly decreased serum lipase levels. With these results, it was argued that esomeprazole could cause a significant decrease in serum amylase and lipase levels in rats with acute pancreatitis.

In conclusion, histopathological evaluation showed that esomeprazole could reduce tissue damage in both the pancreas and lungs in rats with acute pancreatitis. In addition, it was seen that esomeprazole could significantly decrease serum IL-β, TNF-α, amylase, and MIP-2 levels and thus produce an anti-inflammatory effect. However, it was argued that further studies are needed to detail the mechanisms of this effect.

### Limitations

The study had some limitations. *First*, this study included the findings obtained 24 h after the induction of acute pancreatitis. Therefore, this study did not contain data on subacute and chronic processes. *Secondly*, the effects of esomeprazole in acute pancreatitis were not compared with the effects of other pharmacologic agents in this study. *Finally*, further histopathological (such as immunohistochemistry, electron microscopy) and biochemical (such as Western blot) analysis methods that could reveal the anti-inflammatory, antioxidant, and possible anti-apoptotic, anti-ferroptosis, and anti-autophagic mechanisms of action of esomeprazole in detail were not included in this study due to technical or financial restrictions. However, this study’s results demonstrated that esomeprazole could have anti-inflammatory effects on acute pancreatitis. Therefore, this study is preliminary. In light of these results, it was concluded that further studies with esomeprazole treatment protocols in acute pancreatitis and acute lung injury secondary to pancreatitis are needed.

## 5. Conclusions

This study showed that esomeprazole could have therapeutic effects on acute pancreatic and lung damage secondary to pancreatitis in rats. Therefore, it was suggested that esomeprazole could be preferred in clinical practice during acute pancreatitis treatment modalities. Moreover, the results of this study could serve as a basis for future research, and therefore, it could be envisaged that new treatment protocols for acute pancreatitis and related acute lung injury can be developed using different doses, experimental models, and subject numbers.

## Figures and Tables

**Table 1:** Descriptive table of histopathologic examination score results of pancreas and lung tissue of the treatment groups.

	Variable	CONTROL	PCT	ESM	X^2^	*p*
		Mean ± SD/ Median (Min–Max)	Mean ± SD/ Median (Min–Max)	Mean ± SD/ Median (Min–Max)		
Pancreas	Edema	0 (0–0)	2 (2–3)	2 (2–1)	19.413	<0.001
	Inflammation	0 (0–0)	3 (2–3)	2 (2–3)	18.688	<0.001
	Vacuolization	0 (0–0)	4 (3–4)	2 (2–3)	21.445	<0.001
	Necrosis	0 (0–0)	3 (2–3)	2 (1–2)	19.413	<0.001
Lung	Alveolar edema	0 (0–0)	4 (3–4)	2 (2–3)	20.034	<0.001
	Alveolar distention	0 (0–0)	3 (2–3)	2 (1–2)	19.642	<0.001
	Alveolar wall thickness	0 (0–0)	4 (3–4)	2 (2–3)	20.236	<0.001
	PMNL infiltration	0 (0–0)	4 (3–4)	2 (2–3)	21.018	<0.001

Kruskal–Wallis test; *p* < 0.05. (SD: standard deviation, min: minimum, max: maximum, PMNL: polymorphonuclear leucocyte)

**Table 2:** Pairwise group comparisons revealed that the levels of edema, vacuolization, and necrosis in the pancreas differed among the three groups. In addition, alveolar edema, distension, wall thickness, and PMNL infiltration values in the lung tissue were different among all groups.

	Variable	Group (I/J)	Z	*p*
Pancreas	Edema	CONTROL/PCT	−3.664	<0.001
		CONTROL/ESM	−3.703	<0.001
		PCT/ESM	−2.183	0.029
	Inflammation	CONTROL/PCT	−3.703	<0.001
		CONTROL/ESM	−3.703	<0.001
		PCT/ESM	−1.936	0.053
	Vacuolization	CONTROL/PCT	−3.651	<0.001
		CONTROL/ESM	−3.771	<0.001
		PCT/ESM	−3.371	0.001
	Necrosis	CONTROL/PCT	−3.664	<0.001
		CONTROL/ESM	−3.703	<0.001
		PCT/ESM	−2.183	0.029
Lung	Alveolar edema	CONTROL/PCT	−3.664	<0.001
		CONTROL/ESM	−3.664	<0.001
		PCT/ESM	−2.805	0.005
	Alveolar distention	CONTROL/PCT	−3.651	<0.001
		CONTROL/ESM	−3.664	<0.001
		PCT/ESM	−2.578	0.010
	Alveolar wall thickness	CONTROL/PCT	−3.651	<0.001
		CONTROL/ESM	−3.664	<0.001
		PCT/ESM	−2.922	0.003
	PMNL infiltration	CONTROL/PCT	−3.664	<0.001
		CONTROL/ESM	−3.703	<0.001
		PCT/ESM	−3.229	0.001

Mann–Whitney U test, *p* < 0.05. (PMNL: polymorphonuclear leucocyte).

**Table 3:** Descriptive table of pancreas and lung tissue biochemical analysis and serum biochemical analysis results of treatment groups.

Variable	CONTROL	PCT	ESM	F/X^2^	*p*
	Mean ± SD/ Median (Min–Max)	Mean ± SD/ Median (Min–Max)	Mean ± SD/ Median (Min–Max)		
Streptolysin-O (pancreas)	3.64 ± 0.29	5.00 ± 0.48	4.83 ± 0.48	24.516 *	<0.001
Streptolysin-O (lung)	6.10 ± 0.35	7.77 ± 0.59	7.52 ± 0.56	24.886 *	<0.001
Myeloperoxidase (pancreas)	1.65 ± 0.52	2.39 ± 0.58	2.25 ± 0.69	3.428 *	0.051
Myeloperoxidase (lung)	2.01 ± 0.59	2.90 ± 0.71	2.73 ± 0.69	4.008 *	0.034
Nitric oxide (pancreas)	3.64 ± 0.29	5.01 ± 0.48	4.82 ± 0.44	25.873 *	<0.001
Nitric oxide (lung)	6.10 ± 0.35	7.77 ± 0.59	7.47 ± 0.53	25.144 *	<0.001
Interleukin 1β (serum)	13.08 ± 2.04	27.99 ± 4.59	23.30 ± 3.09	40.137 *	<0.001
TNF-α (serum)	48.38 ± 7.57	103.55 ± 16.97	86.22 ± 11.43	40.132 *	<0.001
MIP.2 (serum)	134.11 (75.26–160.85)	2853.96 (1167.43–5180.15)	596.68 (476.18–1782.67)	19.245 †	<0.001
ICAM.1 (serum)	3.13 ± 1.30	16.87 ± 6.70	14.27 ± 6.62	14.312 *	<0.001
Amylase (serum)	3.20 ± 1.33	10.51 ± 2.44	6.95 ± 1.66	30.333 *	<0.001
Lipase (serum)	71.30 (34.28–92.77)	890.51 (432.98–1084.88)	476.52 (350.76–1078.88)	16.141 †	<0.001

* F value, One-Way Analysis of Variance test; ^†^ X^2^ value, Kruskal–Wallis test; *p* < 0.05. (SD: standard deviation, min: minimum, max: maximum, TNF: tumor necrosis factor, ICAM: intercellular adhesion molecule, MIP: macrophage inflammatory protein).

**Table 4:** Paired group comparisons showed that esomeprazole significantly decreased serum IL-β, TNF-α, MIP-2, and amylase levels.

Variable	Group (I/J)	Mean Difference/Z	*p*
Streptolysin-O (pancreas)	CONTROL/PCT	−1.370 *	<0.001
	CONTROL/ESM	−1.191 *	<0.001
	PCT/ESM	0.179 *	0.696
Streptolysin-O (lung)	CONTROL/PCT	−1.673 *	<0.001
	CONTROL/ESM	−1.424 *	<0.001
	PCT/ESM	0.249 *	0.619
Myeloperoxidase (lung)	CONTROL/PCT	−0.888 *	0.037
	CONTROL/ESM	−0.725 *	0.100
	PCT/ESM	0.163 *	0.877
Nitric oxide (pancreas)	CONTROL/PCT	−1.370 *	0.001
	CONTROL/ESM	−1.180 *	0.001
	PCT/ESM	0.190 *	0.634
Nitric oxide (lung)	CONTROL/PCT	−1.672 *	<0.001
	CONTROL/ESM	−1.373 *	<0.001
	PCT/ESM	0.299 *	0.472
Interleukin 1β (serum)	CONTROL/PCT	−14.910 *	<0.001
	CONTROL/ESM	−10.225 *	<0.001
	PCT/ESM	4.685 *	0.031
Tumor necrosis factor-α (serum)	CONTROL/PCT	−55.167 *	<0.001
	CONTROL/ESM	−37.832 *	<0.001
	PCT/ESM	17.335 *	0.031
MIP.2 (serum)	CONTROL/PCT	−3.361 †	0.001
	CONTROL/ESM	−3.240 †	0.001
	PCT/ESM	−3.125 †	0.002
ICAM−1 (serum)	CONTROL/PCT	−13.739 *	<0.001
	CONTROL/ESM	−11.141 *	0.002
	PCT/ESM	2.598 *	0.631
Amylase (serum)	CONTROL/PCT	−7.315 *	<0.001
	CONTROL/ESM	−3.751 *	0.003
	PCT/ESM	3.565 *	0.004
Lipase (serum)	CONTROL/PCT	−3.361 †	0.001
	CONTROL/ESM	−3.240 †	0.001
	PCT/ESM	−1.620 †	0.105

* mean difference value. Tukey Multiple Comparisons test; † Z value. Mann Whitney U test; *p* < 0.05. (ICAM: Intercellular Adhesion Molecule. MIP: Macrophage Inflammatory Protein, TNF: tumor necrosis factor).

## Data Availability

For confidentiality reasons, the data cannot be accessed by third parties.
